# Supplementary material for: The Gain-of-Function Mutation, OsSpl26, Positively Regulates Plant Immunity in Rice
Source: Int J Mol Sci. 2022 Nov 16;23(22):14168. doi: 10.3390/ijms232214168 (PMC9697700; doi:10.3390/ijms232214168)
Supplement: Supplementary file 1 [file ijms-23-14168-s001.zip › ijms-2013966-supplementary.pdf]

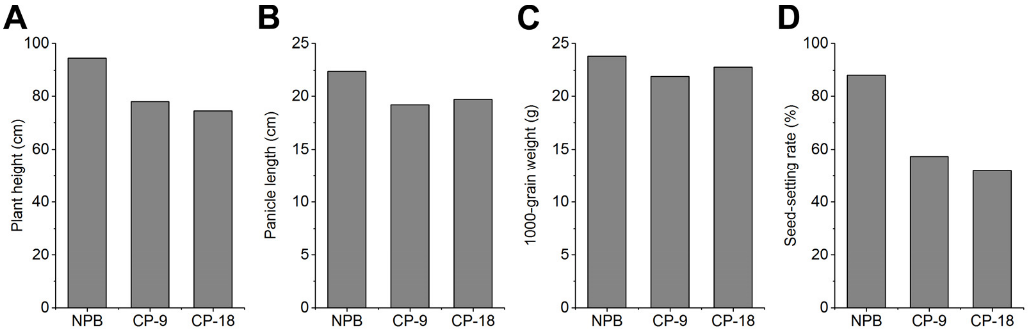

Figure S1. Performance of major agronomic traits.
Major agronomic traits of NPB and reverse complementary lines CP-9 and CP-18 at the heading stage. **(A)** Plant height; **(B)** Panicle length; **(C)** 1000-grain weight; **(D)** Seed-setting rate. Data are from single individual plant.

Table S1. The primer pairs used in this study

Name	Sequence (5′→3′)	Purpose
DNA-1-F	CCATGATTACGAATTAAACTCCCCCAAACATCTTCTGAAA	Construction
DNA-1-R	TACCGAGCTCGAATTGAATTCTGGCAGCCAACACATTC	
DNA-2-F	GGAGGCGAGAGGAATGTGTTGG	Construction
DNA-2-R	GGATCCATGCTAGGGGACGGGATATC	
U3-F	GGCACGGCGACCGTGAGGCCGTT	Construction
U3-R	AAACAACGGCCTCACGGTCGCCG	
U6a-F	GCCGGGATTAGCAGACATGTTGG	Construction
U6a-R	AAACCCAACATGTCTGCTAATCC	
U-F	CTCCGTTTTACCTGTGGAATCG	Construction
gRNA-R	CGGAGGAAAATTCCATCCAC	
Uctcg-B1′	TTCAGAggtctcTctcgCACTGGAATCGGCAGCAAAGG	Construction
gRctga-B2	AGCGTGggtctcGtcagGGTCCATCCACTCCAAGCTC	
Uctga-B2′	TTCAGAggtctcTctgaCACTGGAATCGGCAGCAAAGG	Construction
gRcggt-BL	AGCGTGggtctcGaccgGGTCCATCCACTCCAAGCTC	
GusEcoR1-F	CGGAATTCACAGACAAAACTTGGCGTTGTTG	Construction

GusBamH1-R	CGGGATCCGTTGGTGGATGAATAATGAATTACTGCT	
PANXba1-F	GCTCTAGAATGTCTGCTAATCCCAACCA	Construction
PANBamH1-R	CGGGATCCACAATGTAATGATCCAATTGGAATGAT	
QPCR-F	TTCGACGATTTCAACAAGCAAGCCT	qRT-PCR
QPCR-R	TACACGAGGTAAACAATGGCGACT	
Ubiquitin F	CCCTCCACCTCGTCCTCAG	qRT-PCR
Ubiquitin R	AGATAACAACGGAAGCATAAAAGTC	
PR10-F	CACCATCTACACCATGAAGC	qRT-PCR
PR10-R	AGCACATCCGACTTTAGGAC	
LOX-F	GATGGCGGTGCTCGACGTGCT	qRT-PCR
LOX-R	GCACCTGTTCTTGAGCTTTCTAT	
AOS2-F	CTCGTCGGAAGGCTGTTGCT	qRT-PCR
AOS2-R	ACGATTGACGGCGGAGGTT	
Jamyb-F	CCGAGCATGGTGACTAGCTCATCTT	qRT-PCR
Jamyb-R	CCTTGCACCCAACCGTTAAGCTGTT	
WRKY45-F	TTCCTTGTTGATGTGTCGTCTCA	qRT-PCR
WRKY45-R	CCCCCAGCTCATAATCAAGAAC	
NPR1-F	GGCAGGTGAGAGTCTACGAGGAA	qRT-PCR
NPR1-R	GCTGTCATCCGAGCTAAGTGTT	
EDS1-F	CATTCCAAGAACGAGGACACTG	qRT-PCR
EDS1-R	CAAGACTCAAGGCTAGAACCGA	
PAL3-F	CGCTGAGGCGTTTAAGATTG	qRT-PCR
PAL3-R	GGCAAGGACAGCAAGAATG	
PAL4-F	CTTCACAACAGCTAATCGAG	qRT-PCR
PAL4-R	CGCACTCCATTTCAGTACCA	
